# Immunogenicity, Safety, and Clinical Outcomes Following Homologous and Heterologous SARS-CoV-2 Vaccination Regimens in Patients with Axial Spondyloarthritis and Psoriatic Arthritis: A Prospective Observational Study from the SAFER Cohort

**DOI:** 10.3390/vaccines14100913

**Published:** 2026-10-20

**Authors:** Vanessa de Oliveira Magalhães, Priscila Dias Cardoso Ribeiro, Flávia Maria Matos Melo Campos Peixoto, Erika Biegelmeyer, Mariana Freitas de Aguiar, Cristiane Kayser, Alexandre Wagner Silva de Souza, Charlles Heldan de Moura Castro, Ketty Lysie Libardi Lira Machado, José Geraldo Mill, Nésio Fernandes de Medeiros Junior, Danielle Cristina Filgueira Alves Batista, Rebeca Silva Moreira da Fraga, Thaisa de Moraes Ribeiro Espirito Santo, Samira Tatiyama Miyamoto, Gabriel Smith Sobral Vieira, Pietra Zava Lorencini, Bárbara Oliveira Souza, Heitor Filipe Surlo, Anna Carolina Simões Moulin, Paula dos Santos Athayde, Mariana de Oliveira Macabú, Sandra Lúcia Euzébio Ribeiro, Raquel Lima de Lima, Natália Seixas de Melo, Russian Teixeira Rebello, Marcos Mavignier Sousa Dias, Jonathan Willian da Silva Rodrigues, Camila Maria Paiva França Telles, Natália Sarzi Sartori, Regiane Lima, Rodrigo Poubel Vieira de Rezende, Katia Lino Baptista, Rodrigo Cutrim Gaudio, Ana Karla Guedes de Melo, Valéria Bezerra da Silva, Vitor Alves Cruz, Jozelia Rêgo, Rejane Maria Rodrigues de Abreu Vieira, Adah Sophia Rodrigues Vieira, Adriana Maria Kakehasi, Anna Carolina Faria Moreira Gomes Tavares, Artur José Azevedo Pereira, Renata Henriques de Azevedo, Valderilio Feijó Azevedo, Olindo Assis Martins-Filho, Vanessa Peruhype-Magalhães, Emilia Inoue Sato, Ricardo Machado Xavier, Andréa Teixeira-Carvalho, Viviane Angelina de Souza, Gilda Aparecida Ferreira, Edgard Torres dos Reis-Neto, Odirlei André Monticielo, Valéria Valim, Gecilmara Salviato Pileggi, Marcelo de Medeiros Pinheiro

**Affiliations:** 1 Escola Paulista de Medicina (EPM), Universidade Federal de São Paulo (UNIFESP), Sao Paulo 04023-062, SP, Brazil; 2 Hospital Universitário Cassiano Antônio Moraes, Universidade Federal do Espírito Santo (HUCAM-UFES/EBSERH), Vitoria 29041-295, ES, Brazil; 3 Faculdade de Medicina, Universidade Federal do Amazonas (UFAM), Manaus 69067-005, AM, Brazil; 4 Serviço de Reumatologia, Hospital de Clínicas de Porto Alegre, Universidade Federal do Rio Grande do Sul (UFRGS), Porto Alegre 90035-903, RS, Brazil; 5 Department of Rheumatology, Faculdade de Medicina, Universidade Federal Fluminense (UFF), Niteroi 24020-140, RJ, Brazil; 6 Hospital Universitário Lauro Wanderley, Universidade Federal da Paraíba (UFPB), Joao Pessoa 58051-900, PB, Brazil; 7 Faculdade de Medicina, Universidade Federal de Goiás (UFG), Goiânia 74690-900, GO, Brazil; 8 Hospital Geral de Fortaleza (HGF), Universidade de Fortaleza (UNIFOR), Fortaleza 60811-905, CE, Brazil; 9 Departamento do Aparelho Locomotor, Faculdade de Medicina, Universidade Federal de Minas Gerais (UFMG), Belo Horizonte 30130-100, MG, Brazil; 10 Faculdade de Medicina, Universidade Federal de Juiz de Fora (UFJF), Juiz de Fora 36036-900, MG, Brazil; 11 Departamento de Clínica Médica, Setor de Ciências da Saúde, Universidade Federal do Paraná (UFPR), Curitiba 80060-900, PR, Brazil; 12 Instituto René Rachou, Fundação Oswaldo Cruz (Fiocruz Minas), Belo Horizonte 30190-009, MG, Brazil

**Keywords:** SARS-CoV-2, COVID-19 vaccine, heterologous vaccination, axial spondyloarthritis, psoriatic arthritis, immunogenicity, vaccine safety, immune-mediated rheumatic diseases

## Abstract

Background/Objectives: Patients with spondyloarthritis (SpA) frequently receive immunomodulatory therapies that may influence vaccine-induced immune responses. However, evidence comparing the immunogenicity, safety, and clinical outcomes following homologous and heterologous SARS-CoV-2 vaccination regimens in axial spondyloarthritis (axSpA) and psoriatic arthritis (PsA) remains limited. Methods: This observational study was conducted within the Brazilian multicenter SAFER cohort and included adults with axSpA or PsA who received three doses of SARS-CoV-2 vaccines through the Brazilian National Immunization Program. The primary outcome was humoral immunogenicity, assessed by anti-receptor-binding domain (anti-RBD) IgG geometric mean titers (GMTs). Clinical outcomes, disease activity, and safety were evaluated throughout follow-up. Results: Ninety patients (axSpA = 69; PsA = 21) were included. Antibody titers increased progressively after vaccination, with GMTs rising from 6.70 BAU/mL at baseline to 907.72 BAU/mL after the additional dose. Humoral responses were comparable between axSpA and PsA throughout follow-up. Participants with previous SARS-CoV-2 exposure consistently exhibited higher antibody responses than infection-naïve individuals. Although ChAdOx1-S elicited stronger early humoral responses than CoronaVac, heterologous regimens incorporating a BNT162b2 additional dose produced the highest post-additional-dose antibody concentrations. Serological response rates reached 100% after the additional dose, irrespective of disease subtype or vaccination regimen. Biologic disease-modifying antirheumatic drugs (bDMARDs), including TNF inhibitors, were not significantly associated with reduced antibody responses. Only four COVID-19 cases occurred during follow-up, with no hospitalizations or deaths. Disease activity remained stable, and adverse events were predominantly mild and self-limited. Conclusions: SARS-CoV-2 vaccination was safe and highly immunogenic in patients with axSpA and PsA, without compromising disease activity control. Heterologous vaccination regimens incorporating a BNT162b2 additional dose elicited the strongest humoral immune responses. These findings provide prospective real-world evidence supporting optimized additional-dose strategies and may inform future vaccination approaches for patients with SpA.

## 1. Introduction

Coronavirus disease 2019 (COVID-19), caused by severe acute respiratory syndrome coronavirus 2 (SARS-CoV-2), emerged in late 2019 and rapidly evolved into a global public health emergency. In March 2020, the World Health Organization (WHO) declared COVID-19 a pandemic. Since the emergence of SARS-CoV-2, more than 779 million confirmed COVID-19 cases and over 7 million deaths had been reported worldwide to the World Health Organization as of 28 June 2026. These figures reflect reported cases and deaths and likely underestimate the true burden of infection, as SARS-CoV-2 continues to circulate amid changes in testing and surveillance practices. Brazil was among the countries severely affected during the early pandemic waves, with high hospitalization and mortality rates [1,2].

The rapid development of SARS-CoV-2 vaccines represented a major turning point in the control of the pandemic and introduced multiple vaccine platforms, including inactivated whole-virus, adenoviral vector, and messenger RNA (mRNA) vaccines. These technologies differ in their mechanisms of antigen presentation and immune activation, resulting in distinct humoral and cellular immune responses. As evidence accumulated during the pandemic, heterologous vaccination schedules combining different vaccine platforms emerged as an effective strategy to enhance the magnitude and durability of vaccine-induced immunity, particularly in immunocompromised populations [3,4,5,6].

In Brazil, the national vaccination campaign began in January 2021 following the emergency authorization of CoronaVac (Sinovac/Butantan), an inactivated whole-virus vaccine, and ChAdOx1-S (Vaxzevria/Oxford/AstraZeneca–Fiocruz), a chimpanzee adenoviral vector vaccine. Subsequently, the BNT162b2 (Comirnaty; Pfizer-BioNTech), an mRNA vaccine, was incorporated into the Brazilian National Immunization Program (NIP), enabling both homologous and heterologous strategies, as well as additional dose strategies. The expansion of vaccine coverage was associated with substantial reductions in severe COVID-19, hospitalization, and mortality, reinforcing vaccination as the cornerstone of pandemic control [7,8].

Spondyloarthritis (SpA), encompassing axial spondyloarthritis (axSpA) and psoriatic arthritis (PsA), is a group of chronic inflammatory diseases that frequently require long-term immunomodulatory therapy. As these patients may be at increased risk of adverse COVID-19 outcomes and frequently receive biologic disease-modifying antirheumatic drugs (bDMARDs) or targeted synthetic disease-modifying antirheumatic drugs (tsDMARDs), vaccination rapidly became a key component of disease management [9]. Current American College of Rheumatology (ACR) recommendations support the administration of non-live vaccines in patients with immune-mediated rheumatic diseases (IMRDs) regardless of disease activity [10,11]. Given these characteristics, understanding the immunogenicity, safety, and clinical outcomes associated with different SARS-CoV-2 vaccination strategies in patients with SpA has become an important clinical priority.

Accumulating evidence indicates that COVID-19 vaccines are generally safe and effective in patients with SpA [4,11,12]. Most patients develop adequate humoral and cellular immune responses following vaccination, and no consistent increase in disease flares has been observed. Nevertheless, vaccine-induced immunity may be influenced by several factors, including previous SARS-CoV-2 exposure and the use of immunosuppressive therapies. TNF inhibitor (TNFi) treatment has been associated with lower antibody titers and a more rapid decline in humoral responses, although protection against severe disease appears to be maintained [13]. In addition, Aikawa et al. demonstrated that the frequency of anti-SARS-CoV-2 antibody positivity increased from 60% to 93% after a third dose of CoronaVac, highlighting the importance of additional-dose vaccination in patients with IMRDs [5].

Studies specifically evaluating SARS-CoV-2 vaccination in patients with SpA remain limited. Saad et al. demonstrated reduced humoral immunogenicity after two doses of CoronaVac in patients with SpA compared with healthy controls, particularly among individuals receiving TNFi, methotrexate (MTX), or glucocorticoids (GC) [11]. Importantly, administration of an additional dose substantially enhanced both antibody and neutralizing responses, highlighting the value of additional vaccine doses in this population [14].

Consistent with these findings, Silva et al. reported a progressive decline in humoral immunity six months after CoronaVac vaccination in patients with IMRDs, particularly among males and individuals receiving GC, TNFi, abatacept, or rituximab (RTX), emphasizing the need to evaluate the durability of vaccine-induced immunity and the impact of additional dose strategies [6]. In addition, Marques et al. observed that SARS-CoV-2 infection was not associated with worsening disease activity in patients with SpA and that HLA-B27 status did not confer protection against infection, providing further insight into the clinical impact of COVID-19 in this population [9].

Although previous studies have demonstrated that SARS-CoV-2 vaccines are generally safe and highly immunogenic in patients with rheumatic diseases, several clinically relevant questions remain unanswered. Few prospective longitudinal studies have directly compared different vaccine platforms and homologous versus heterologous vaccination regimens in patients with axSpA and PsA under real-world conditions. Moreover, the influence of previous SARS-CoV-2 exposure, bDMARDs, and additional dose strategies on the magnitude and durability of humoral immune responses and disease activity has not been fully characterized [15].

To address these knowledge gaps, we conducted a prospective longitudinal study within the Brazilian multicenter SAFER (**SAF**ety, **E**ffectiveness, and Du**R**ation of immunity after SARS-CoV-2 vaccination in patients with IMRDs cohort), which includes patients with systemic lupus erythematosus (SLE), vasculitis, rheumatoid arthritis (RA), and SpA [16,17,18].

The present study aimed to compare the immunogenicity, safety, occurrence of COVID-19 during follow-up, and effects on disease activity of homologous and heterologous SARS-CoV-2 vaccination regimens based on inactivated whole-virus (CoronaVac), adenoviral vector (ChAdOx1-S), and mRNA (BNT162b2) platforms in a real-world cohort of patients with axSpA and PsA.

## 2. Materials and Methods

### 2.1. Study Design and Population

This prospective, multicenter, longitudinal observational study was conducted within the SAFER cohort, a nationwide registry established to evaluate the immunogenicity, safety, and clinical outcomes of SARS-CoV-2 vaccination in patients with IMRDs. Vaccination regimens were not assigned by the investigators. As SAFER was an observational cohort rather than an interventional clinical trial, registration on ClinicalTrials.gov was not required.

Participants were recruited from tertiary referral centers located in 10 Brazilian states, encompassing all five geographic regions of Brazil. They were prospectively followed from June 2021 to March 2024, with the final in-person assessment performed four months after additional dose vaccination. Laboratory and disease activity analyses were performed through T4, the last in-person study visit. Subsequent follow-up (T5) was conducted remotely through telephone calls, text messages, and document exchange solely to ascertain incident COVID-19 cases. No laboratory or disease activity assessments were performed at T5.

**Figure 1:**
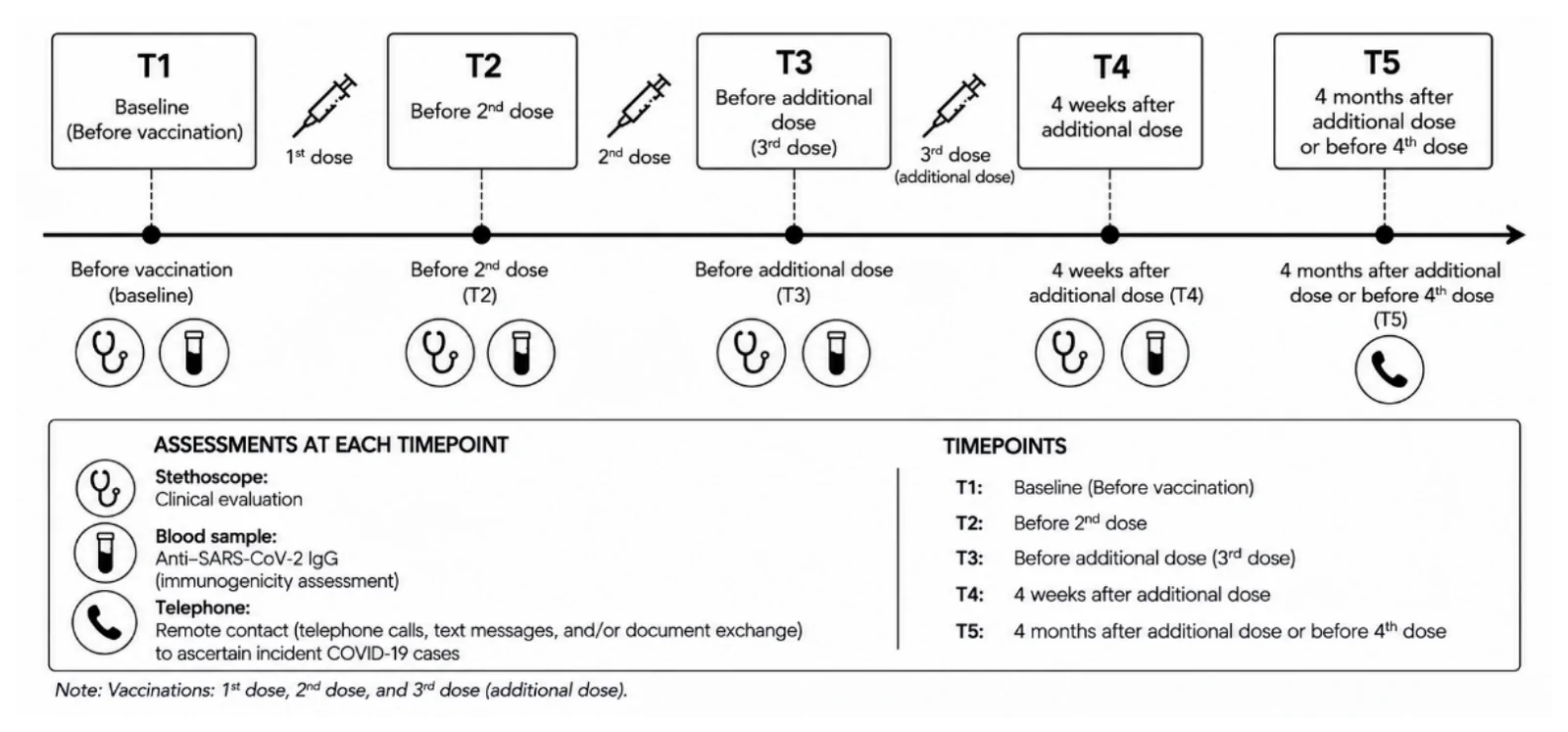
Study timeline of SARS-CoV-2 vaccination, clinical evaluations, and anti-SARS-CoV-2 IgG measurements in patients with axial spondyloarthritis and psoriatic arthritis.

Vaccination schedules were determined exclusively by the NIP according to vaccine availability and national recommendations, without investigator intervention. This real-world prospective cohort included consecutive adult patients with axSpA or PsA who fulfilled the eligibility criteria. Given the observational design, no formal sample size calculation was performed. All eligible participants with complete longitudinal immunogenicity data were included in the final analysis (Figure 1).

Eligible participants were aged ≥18 years and had a rheumatologist-confirmed diagnosis of axSpA or PsA fulfilling the assessment of SpondyloArthritis International Society (ASAS) classification criteria for axSpA and the Classification Criteria for Psoriatic Arthritis (CASPAR), respectively [19,20]. All participants provided written informed consent before enrollment.

Participants were excluded if they had a history of severe vaccine-related adverse reactions; active human immunodeficiency virus (HIV) infection with CD4 counts <200 cells/mm^3^; active hepatitis B or C infection; malignancy within the previous five years except non-melanoma skin cancer; pregnancy; a history of solid-organ transplantation; hematologic disorders; or primary immunodeficiency syndromes.

### 2.2. Clinical and Treatment Variables

Demographic and clinical data included age, sex, ethnicity/skin color, body mass index (BMI), smoking status, alcohol consumption, disease subtype, disease duration, HLA-B27 status, extramusculoskeletal manifestations, and relevant comorbidities, including hypertension, diabetes mellitus, and cardiovascular disease.

Current medications were recorded at baseline and included nonsteroidal anti-inflammatory drugs (NSAIDs), GC, conventional synthetic disease-modifying antirheumatic drugs (csDMARDs), bDMARDs, and tsDMARDs. Participants were classified according to the degree of immunosuppression based on their baseline treatment regimen, following the recommendations of the Brazilian Society of Rheumatology (Table 1) [21].

### 2.3. Vaccination Regimens

Vaccines were administered according to NIP, based on vaccine availability and national recommendations, based on vaccine availability and national recommendations, without investigator intervention or allocation. The vaccines included CoronaVac, ChAdOx1-S, and BNT162b2. All vaccines were the original monovalent formulations encoding or containing the ancestral SARS-CoV-2 spike antigen; no variant-adapted formulations were administered during the study vaccination period [8].

Vaccination schedules were classified as homologous when all doses involved the same vaccine platform and heterologous when different vaccine platforms were combined throughout the vaccination schedule.

The primary vaccination period was defined as the interval from 15 days after the first dose until administration of the additional dose. The post-additional dose period began 15 days after administration of the additional dose and continued until receipt of a fourth vaccine dose or up to 120 days after the additional dose, whichever occurred first (Figure 1).

To minimize potential interference with vaccine-induced immune responses, vaccination was temporarily postponed in participants with recent RTX exposure, SARS-CoV-2 infection, blood product transfusion, plasmapheresis, immunoglobulin administration, cyclophosphamide treatment, glucocorticoid pulse therapy, or recent administration of other vaccines, according to predefined study criteria.

### 2.4. Laboratory Assessment

Humoral immunity was assessed by measuring serum IgG antibodies against the SARS-CoV-2 receptor-binding domain (anti-RBD IgG) using a chemiluminescent microparticle immunoassay (CMIA; SARS-CoV-2 IgG II Quant, (CMIA; SARS-CoV-2 IgG II Quant, Abbott Ireland Diagnostics Division^®^ Sligo, Ireland). Peripheral blood samples were collected at T1 through T4, stored at −80 °C, and analyzed centrally at Fundação Oswaldo Cruz (Fiocruz).

Anti-RBD IgG concentrations were converted from arbitrary units (AU/mL) to binding antibody units (BAU/mL) using a conversion factor of 0.142 (BAU/mL = 0.142 × AU/mL).

Seropositivity was defined as an anti-RBD IgG concentration ≥7.1 BAU/mL. Geometric mean titers (GMTs) were calculated from T1 through T4, and fold increases (FIs) were expressed as ratios of GMTs between consecutive time points (e.g., T2/T1 and T3/T2). As an exploratory analysis, we calculated the proportion of participants with anti-RBD IgG concentrations ≥506 BAU/mL at each time point. In the correlates-of-protection analysis by Feng et al., this threshold was associated with an estimated 80% vaccine efficacy against symptomatic COVID-19. However, because this threshold has not been validated in patients with SpA or for the vaccination regimens evaluated in the SAFER cohort, it was used solely as an exploratory reference and not as a criterion for seropositivity or individual clinical protection [22].

### 2.5. Outcomes

The primary outcome was humoral immunogenicity, assessed through serial measurements of anti-RBD IgG concentrations and GMTs. Secondary outcomes included occurrence of laboratory-confirmed COVID-19 during follow-up, immunogenicity comparisons, vaccine safety, and longitudinal changes in disease activity.

Secondary outcomes included comparisons of immunogenicity across vaccination regimens, the occurrence of confirmed COVID-19 during follow-up, vaccine safety, and longitudinal changes in disease activity.

Safety outcomes comprised local and systemic adverse events (AE) following vaccination (AEFV), serious adverse events (SAEs), and adverse events of special interest (AESIs) [23].

Disease activity was assessed using validated disease-specific instruments, according to disease phenotype and data availability, including the Bath Ankylosing Spondylitis Disease Activity Index (BASDAI) [24], Ankylosing Spondylitis Disease Activity Score (ASDAS) [25], Psoriasis Area and Severity Index (PASI) [26], Minimal Disease Activity (MDA) [27], and Physician Global Assessment (PGA) [28]. Disease activity at T4 was compared with baseline values obtained at T1. Participants were clinically assessed at T1, T2, T3 and T4 (Figure 1). PASI and MDA were assessed exclusively in patients with PsA, whereas BASDAI was assessed in patients with axial disease. ASDAS was calculated only when a recent C-reactive protein (CRP) measurement was available.

### 2.6. Statistical Analysis

Descriptive data are presented as frequencies and percentages or medians and interquartile ranges (IQRs). Anti-RBD IgG concentrations are reported as GMTs with 95% confidence intervals (CIs), and FIs were calculated between consecutive time points. Between-group differences were estimated as GMT ratios (95% CIs) using log-transformed concentrations and Welch’s *t*-test or linear regression, with the Mann–Whitney U test as a sensitivity analysis. Paired comparisons used the Wilcoxon signed-rank test, with Bonferroni correction when appropriate. Within-participant responses were also summarized as the proportion achieving a ≥4-fold increase.

Categorical outcomes were compared using Fisher’s exact test. Immunogenicity proportions are reported with Wilson 95% CIs, and adverse-event risk differences with Newcombe 95% CIs. Univariable and multivariable linear regression models evaluated factors associated with log-transformed anti-RBD IgG concentrations at T4, with back-transformed coefficients reported as adjusted GMT ratios.

Analyses were performed using R 4.2 (tidyverse, gtsummary, and rstatix) and Python 3.13.14 (pandas 2.3.1, NumPy 2.3.1, SciPy 1.18.0, statsmodels 0.14.6, and matplotlib 3.11.0). Python was used for additional GMT-ratio, regression, mixed-effects, and CI analyses. Results from overlapping analyses were cross-checked across environments. Missing data were handled by complete-case analysis. All tests were two-sided, with statistical significance set at *p* < 0.05.

## 3. Results

### 3.1. Study Population

**Figure 2:**
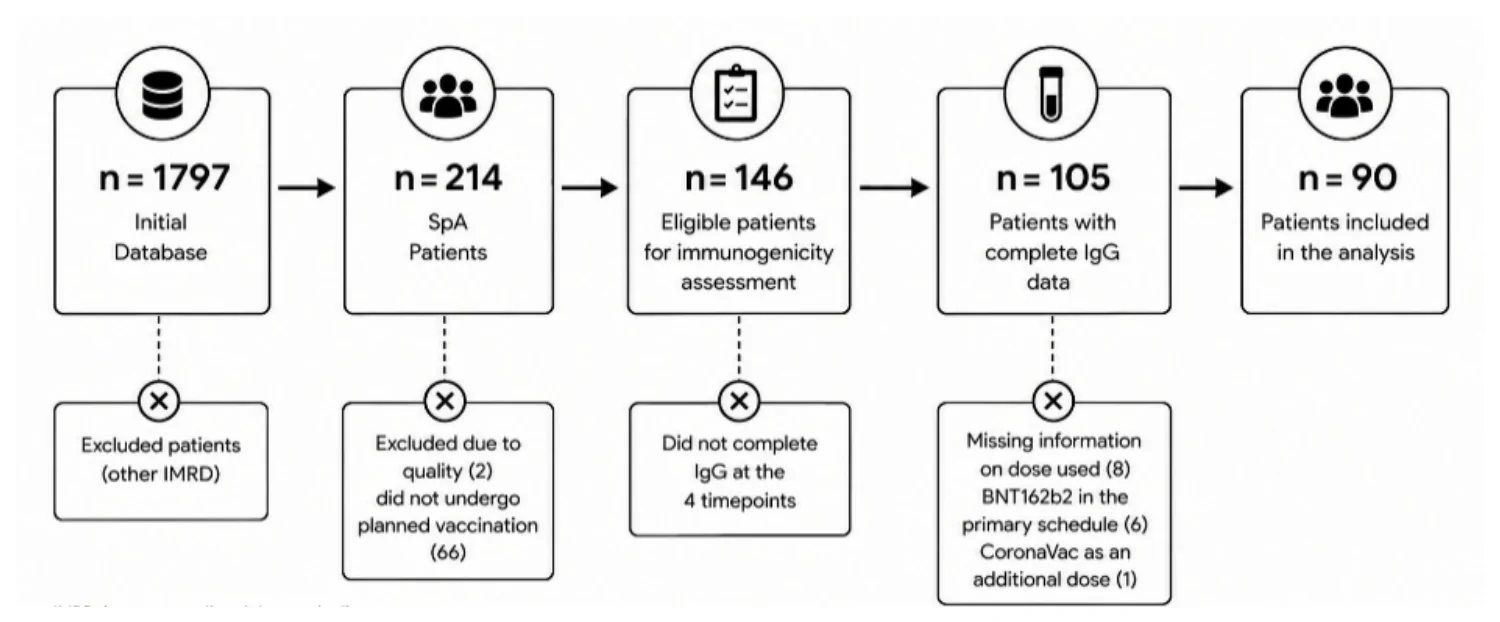
Flowchart of participant selection for the SAFER cohort analysis. IMRD: immune-mediated rheumatic diseases, autoimmune disease; SpA, spondyloarthritis; IgG, immunoglobulin G; BNT162b2, Pfizer–BioNTech COVID-19 vaccine. Arrows indicate participant progression through the study flow, and cross marks indicate exclusions at each stage.

A total of 1821 records were initially identified in the REDCap database. After exclusion of 24 test records, 1797 valid cases of patients with IMRDs remained. Among these, 214 patients had SpA. Following application of the predefined exclusion criteria and removal of participants with incomplete longitudinal immunogenicity assessments, the final study population comprised 90 patients enrolled (Figure 2).

**Figure 3:**
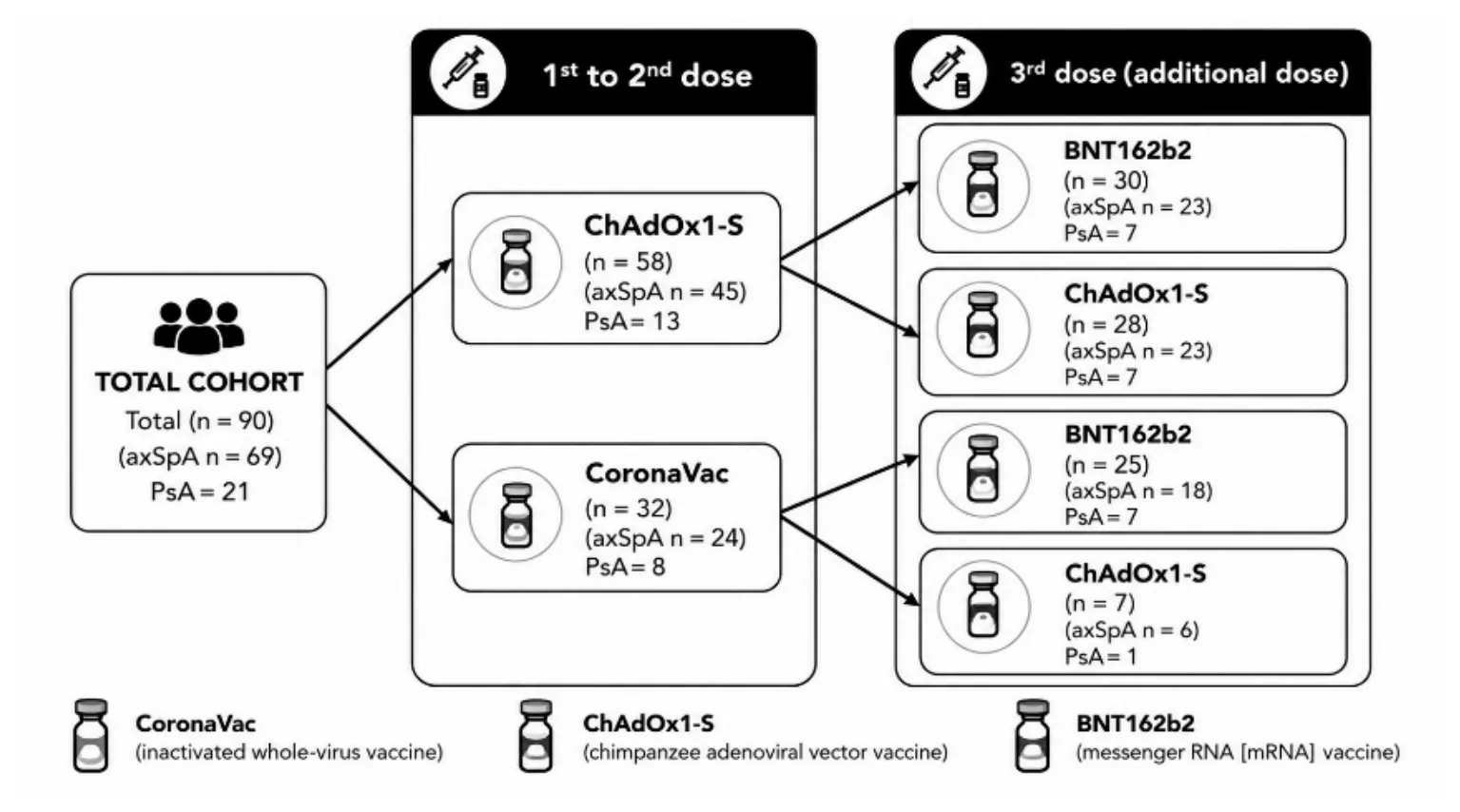
Distribution of primary vaccination and additional dose regimens in the SpA cohort. axSpA, axial spondyloarthritis; PsA, psoriatic arthritis; ChAdOx1-S, chimpanzee adenoviral vector vaccine; CoronaVac, inactivated whole-virus vaccine; BNT162b2, mRNA vaccine.

Vaccines were administered according to availability within the NIP, reflecting routine clinical practice. The distribution of primary vaccination and additional dose regimens is shown in Figure 3.

### 3.2. Baseline Demographic, Clinical, and Treatment Characteristics

Baseline demographic, clinical, and treatment characteristics are summarized in Table 2. Compared with patients with axSpA, those with PsA were older (52 [47–57] vs. 41 [31–49] years, *p* < 0.001), more frequently female (67% vs. 39%, *p* = 0.027), and had a higher BMI (*p* = 0.029). Disease characteristics differed between groups: HLA-B27 positivity was more frequent in axSpA among participants tested (76% vs. 0%, *p* < 0.001), axial involvement predominated in axSpA (91%), and peripheral involvement was more frequent in PsA (76%) (both *p* < 0.001).

MTX use was more frequent in PsA (48% vs. 19%, *p* = 0.02), whereas daily NSAID use was more common in patients with axSpA (22% vs. 0%, *p* = 0.018). No significant differences were observed in the use of bDMARDs, including TNFi, or in skin color, disease duration, previous SARS-CoV-2 infection, and overall immunosuppression level were comparable between the groups.

### 3.3. COVID-19 Occurrence

Overall, 21 laboratory-confirmed COVID-19 cases were documented during follow-up. Two cases occurred between the second and additional vaccine doses, while 17 occurred between T4 and T5, corresponding to 18.9% of the cohort (17/90). Of these 17 cases, 15 occurred in patients with axSpA (15/69, 21.7%) and two in patients with PsA (2/21, 9.5%), with no statistically significant difference between disease subtypes (*p* = 0.340). All 17 post-additional-dose cases occurred between January and February 2022, during the Omicron wave, and before receipt of a fourth vaccine dose. No hospitalizations, persistent sequelae, or deaths were reported.

### 3.4. Humoral Immunogenicity

#### 3.4.1. Humoral Responses According to Disease and Therapy

**Figure 4:**
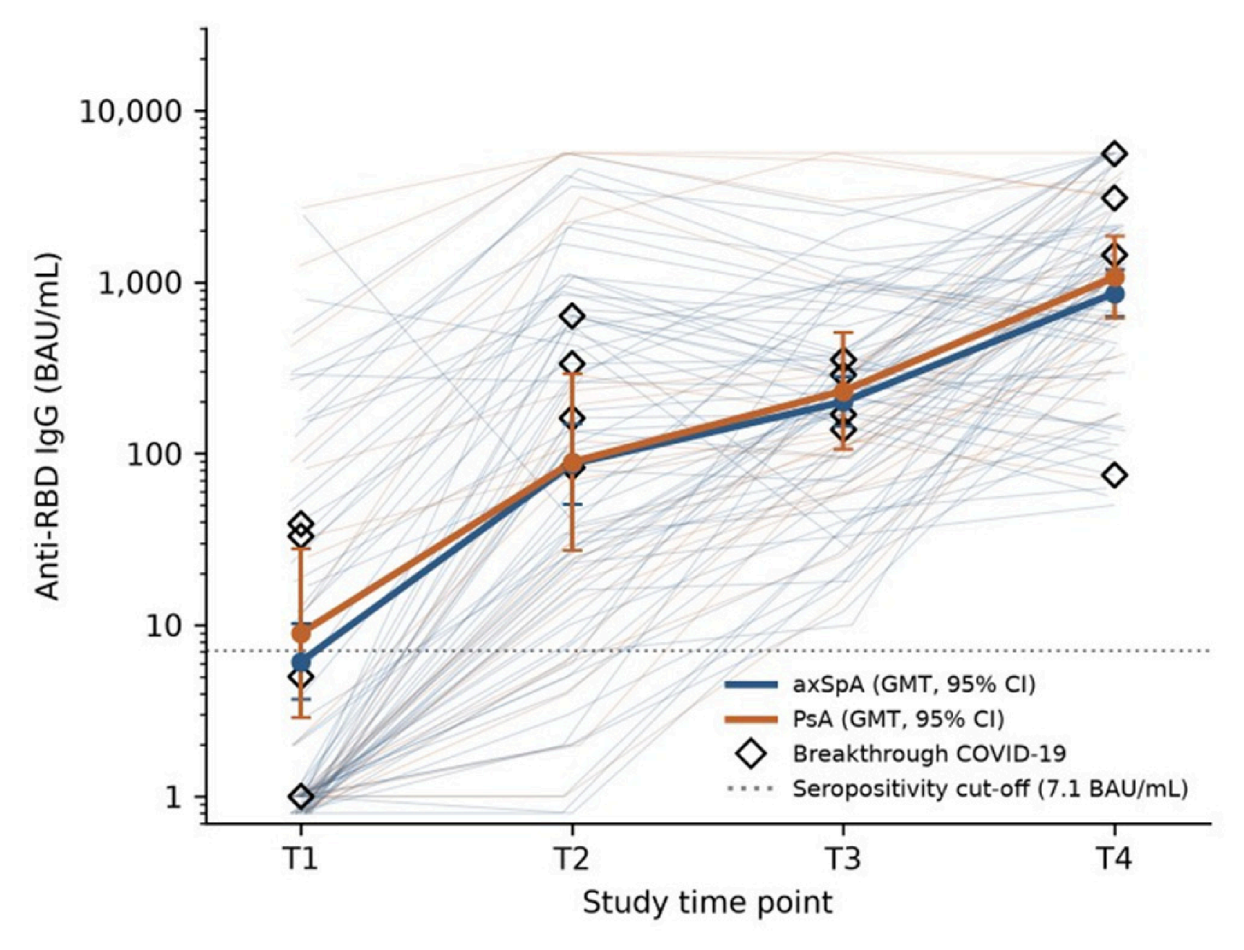
Individual anti-RBD IgG trajectories by disease subtype (axSpA, blue; PsA, orange). axSpA, axial spondyloarthritis; PsA, psoriatic arthritis; GMT, geometric mean titer; CI, confidence interval; RBD, receptor-binding domain; IgG, immunoglobulin G; BAU/mL, binding antibody units per milliliter; T1–T4, study time points.

No significant differences in anti-RBD IgG GMT rates were observed between patients with axSpA and PsA at any study time point. Both disease subgroups exhibited comparable longitudinal antibody kinetics throughout follow-up (Figure 4).

**Figure 5:**
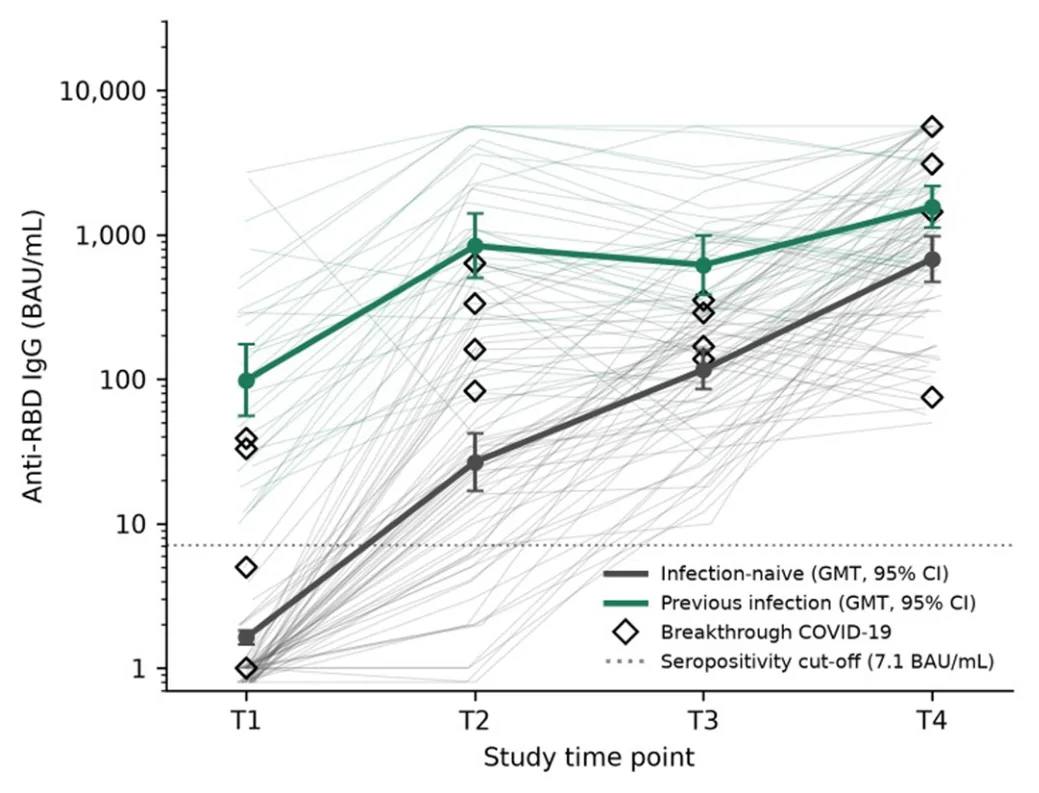
Individual anti-RBD IgG trajectories according to previous SARS-CoV-2 infection status (infection-naïve, gray; previously infected, green). GMT, geometric mean titer; CI, confidence interval; RBD, receptor-binding domain; IgG, immunoglobulin G; BAU/mL, binding antibody units per milliliter; T1–T4, study time points.

**Figure 6:**
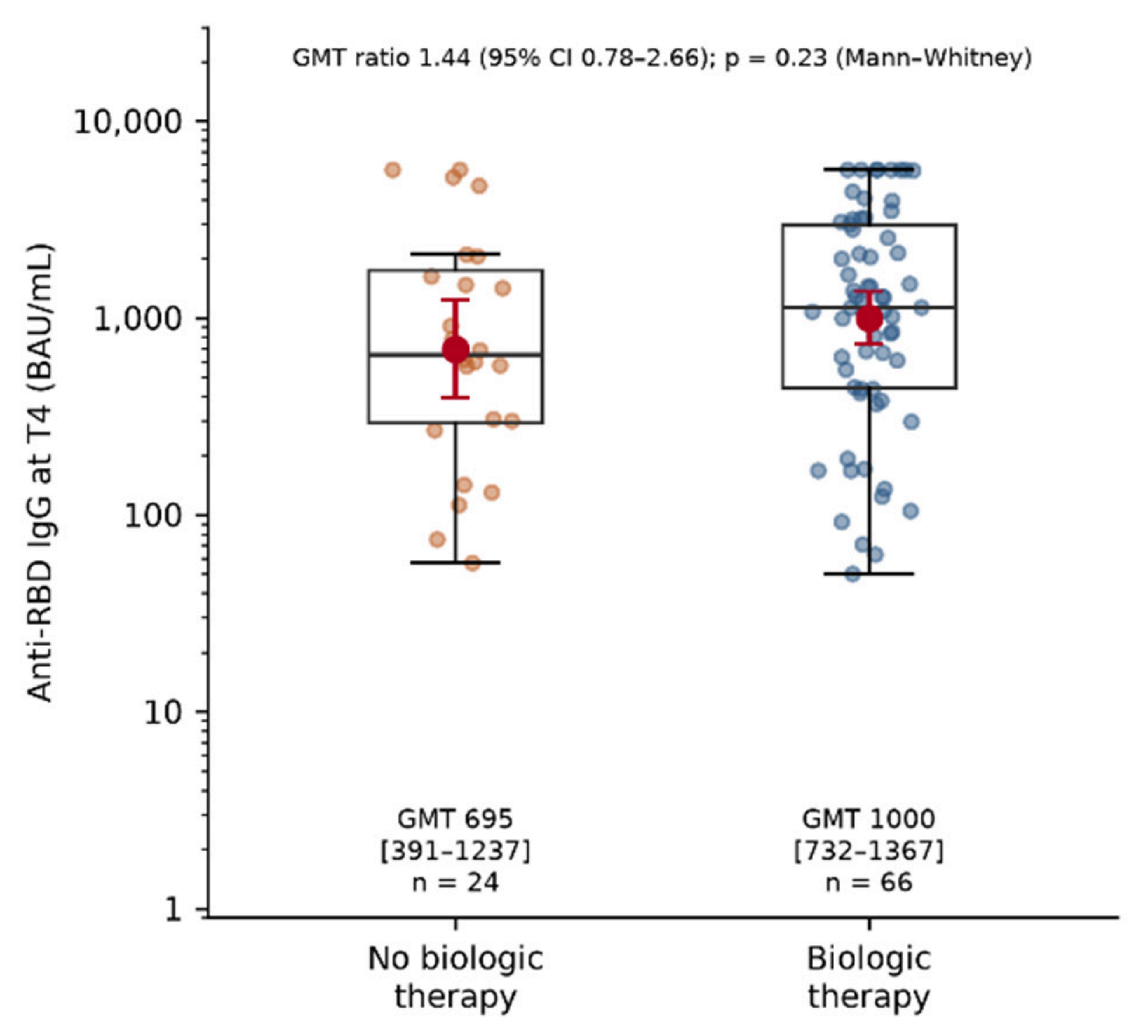
Anti-SARS-CoV-2 RBD IgG antibody levels according to bDMARDs. Each dot represents an individual participant. Boxes indicate the interquartile range, horizontal lines indicate the median, and whiskers show the data distribution. Red dots represent the GMTs. The GMT ratio and corresponding 95% CI compare participants receiving bDMARD therapy with those receiving no bDMARD therapy. Anti-RBD IgG, immunoglobulin G antibodies against the SARS-CoV-2 receptor-binding domain; BAU/mL, binding antibody units per milliliter; CI, confidence interval; GMT, geometric mean titer; T4, post-additional-dose assessment.

Between-group GMT ratios indicated higher anti-RBD IgG concentrations after the ChAdOx1-S primary series than after CoronaVac at T2 and T3. At T4, GMTs were higher among participants who received a BNT162b2 additional dose or a heterologous vaccination regimen. Previous SARS-CoV-2 infection was also associated with higher GMTs, although the magnitude of this difference decreased over time (Figure 5 and Table 3). No statistically significant differences were observed between patients with PsA and axSpA or according to bDMARD use at T4 (Table 3 and Figure 6).

Antibody concentrations are presented in Figure 6 as BAU/mL on a logarithmic scale. Box plots show the median and interquartile range (IQR), whiskers indicate the range, and dots represent individual participants. GMTs were 695 BAU/mL (95% CI: 391–1237) in patients not receiving bDMARD therapy and 1000 BAU/mL (95% CI: 732–1367) in those receiving bDMARD therapy. The between-group GMT ratio was 1.44 (95% CI: 0.78–2.66), with no statistically significant difference between groups (Mann–Whitney U test, *p* = 0.226).

#### 3.4.2. Influence of Previous SARS-CoV-2 Exposure

Participants with previous SARS-CoV-2 infection consistently exhibited higher antibody responses than infection-naïve individuals throughout follow-up (Figure 5).

#### 3.4.3. Humoral Responses According to Primary Vaccination Schedule

Antibody titers increased progressively over time regardless of the primary vaccination regimen. However, participants who received the ChAdOx1-S developed significantly higher antibody titers at T2 and T3 than those vaccinated with CoronaVac (both *p* < 0.001), whereas baseline antibody concentrations were comparable between groups (Table 4).

Serological response rates increased from 34.4% at T1 to 80.0% at T2, reaching 100% at T3 and remaining at 100% at T4. Among participants without previous SARS-CoV-2 infection, the response rate increased from 0% at baseline to 69.5% at T2 and 100% at T3. At T2, serological response rates were 94.8% after the first dose of ChAdOx1-S and 53.1% after CoronaVac. However, all participants were seropositive after completion of the primary series, regardless of disease subtype or vaccination regimen (Table 5).

#### 3.4.4. Humoral Responses According to Additional Dose Strategy

Marked differences emerged after the additional dose. Although the homologous ChAdOx1-S regimen yielded the highest antibody titers immediately before the additional dose (T3), heterologous regimens incorporating a BNT162b2 achieved significantly higher GMT at T4 than homologous regimens (1388.14 [1072.04–1797.44] vs. 354.37 [207.76–604.45] BAU/mL; *p* < 0.001).

The ChAdOx1-S–ChAdOx1-S–BNT162b2 regimen yielded the highest post-additional-dose GMT, whereas the CoronaVac–CoronaVac–BNT162b2 regimen showed the greatest FI between T3 and T4. Similarly, participants receiving a BNT162b2 as an additional dose had significantly higher antibody concentrations at T4 than those receiving a ChAdOx1-S, regardless of the primary vaccination schedule (Table 6).

### 3.5. Longitudinal Assessment of Disease Activity

Among patients with axial involvement, ASDAS and BASDAI remained stable throughout follow-up. No significant differences were observed in ASDAS disease activity categories across study visits (*p* = 0.18), although median ASDAS values decreased from 1.77 [0.97–3.51] at baseline to 1.00 [0.93–1.40] four weeks after the additional dose. Likewise, BASDAI status remained stable throughout follow-up, with no significant differences between visits (*p* = 0.21). Similarly, patients with PsA did not have any significant changes in disease activity over time, either according to PASI categories (*p* = 0.74) or in the proportion of patients achieving MDA (*p* = 0.72).

Longitudinal changes in disease activity are summarized in Table 7.

### 3.6. Factors Associated with Anti-RBD IgG Concentrations After the Additional Dose

Univariable and multivariable regression analyses of anti-RBD IgG concentrations at T4 are presented in Table 8. After adjustment, receipt of a BNT162b2 additional dose (adjusted GMT ratio, 4.34; 95% CI, 2.71–6.95; *p* < 0.001) and previous SARS-CoV-2 infection (adjusted GMT ratio, 3.23; 95% CI, 2.01–5.19; *p* < 0.001) remained significantly associated with higher antibody concentrations. No statistically significant associations were observed for the primary vaccination series, age, disease subtype, or bDMARD therapy. Female sex showed a borderline association (*p* = 0.050) (Table 8). Univariable and multivariable linear regression analyses of factors associated with Anti-RBD IgG concentrations at T4.

### 3.7. Adverse Events

The frequency of local and systemic AEFV is summarized in Table 9. During the primary vaccination series, participants who received ChAdOx1-S reported a higher frequency of injection-site pain (79% vs. 38%, *p* < 0.001), fatigue (53% vs. 25%, *p* = 0.009), and headache (59% vs. 34%, *p* = 0.028) than those who received CoronaVac. In addition, at least one local AE was reported more frequently following ChAdOx1-S than CoronaVac (83% vs. 44%, *p* < 0.001).

Following the additional dose, erythema (15.0% vs. 0%; *p* = 0.038) and swelling (21.0% vs. 3.2%; *p* = 0.042) were more frequently reported among participants receiving BNT162b2 than among those receiving ChAdOx1-S. No other statistically significant differences in local or systemic adverse events were observed between additional-dose regimens, and no vaccine-related SAEs or AESIs were recorded during follow-up.

Absolute risk differences after the primary vaccination series were higher for ChAdOx1-S than for CoronaVac for any local reaction (39.0; 95% CI, 18.5–56.3), injection-site pain (41.8; 95% CI, 20.8–58.6), and fatigue (28.4; 95% CI, 7.2–45.4). Following the additional dose, BNT162b2 was associated with higher absolute risks of erythema (14.9; 95% CI, 1.6–27.7) and swelling (18.1; 95% CI, 2.1–31.9) compared with ChAdOx1-S (Table 9).

## 4. Discussion

This prospective multicenter study provides real-world longitudinal evidence regarding the humoral immunogenicity, safety, occurrence of COVID-19, and disease activity following COVID-19 vaccination in patients with axSpA and PsA, addressing four important unmet needs. First, vaccination elicited robust humoral immune responses across all vaccination regimens. Second, patients with axSpA and PsA exhibited comparable antibody kinetics despite marked demographic and clinical differences. Third, heterologous vaccination strategies incorporating an additional mRNA dose generated substantially stronger humoral responses than homologous schedules. Finally, vaccination was well tolerated, and it was not associated with worsening disease activity, while bDMARDs did not impair vaccine-induced antibody responses.

Patients with SpA maintained robust humoral responses throughout follow-up despite frequent exposure to bDMARDs. These findings are consistent with other studies demonstrating preserved vaccine responsiveness in SpA and extend current evidence by providing longitudinal comparisons across different vaccine platforms and additional dose strategies under routine clinical practice. Unlike diseases requiring more intensive immunosuppression, such as ANCA-associated vasculitis, where B-cell depletion and cyclophosphamide markedly impair vaccine responses, patients with SpA achieved high antibody concentrations after additional dose vaccination, suggesting that treatment-related immunosuppression exerts a greater influence on vaccine responsiveness than the underlying rheumatic disease itself [18].

Previous SARS-CoV-2 exposure was associated with a more robust humoral response to vaccination. Participants with prior infection consistently exhibited higher anti-RBD IgG concentrations throughout follow-up, in keeping with the enhanced immune responses associated with hybrid immunity. Importantly, infection-naïve participants also demonstrated substantial increases in antibody titers following successive vaccine doses, underscoring the value of completing the recommended vaccination schedule regardless of previous infection status.

One of the principal findings of this study was the distinct performance of different vaccine platforms. Although ChAdOx1-S induced stronger antibody responses after the primary vaccination series than CoronaVac, BNT162b2 dose vaccination produced the highest post-additional dose antibody concentrations regardless of the primary schedule. These observations support accumulating evidence that heterologous vaccination strategies induce broader and more potent humoral responses through complementary mechanisms of immune activation.

Our findings expand previous observations from the CoronavRheum study and Saad et al., who demonstrated lower immunogenicity after two doses of CoronaVac in patients with SpA receiving immunosuppressive therapies [14]. By evaluating complete longitudinal vaccination schedules, our study demonstrates that heterologous additional dose strategies, particularly those incorporating BNT162b2, substantially enhance antibody responses and largely overcome the reduced immunogenicity observed after the primary vaccination series.

Importantly, bDMARDs, including TNFi and IL-17i, were not independently associated with diminished humoral responses after additional dose vaccination. Likewise, seroconversion rates approached 100% following the additional dose. These findings agree with previous reports showing that bDMARDs generally preserve vaccine responsiveness, whereas B-cell-depleting therapies and high-dose GC remain the principal determinants of impaired vaccine-induced immunity [4,14,29,30].

The temporal distribution of COVID-19 cases in our cohort warrants particular attention. Although relatively few infections were documented during the earlier vaccination periods, 17 cases (18.9%) occurred between T4 and T5, all between January and February 2022, 65–141 days after the additional dose and before receipt of a fourth vaccine dose. This clustering coincided with the Omicron wave in Brazil and may reflect the emergence of a SARS-CoV-2 variant with greater capacity to evade vaccine-induced immunity. However, the infecting variants were not confirmed by viral sequencing, and the contribution of waning humoral immunity cannot be distinguished from changes in viral circulation or exposure risk. Importantly, no hospitalizations, persistent sequelae, or deaths were reported, although the small number of cases limits conclusions regarding protection against severe COVID-19. These findings highlight the importance of interpreting post-vaccination infections in their epidemiological context and of considering both the timing of vaccination and circulating variants when evaluating the clinical outcomes of additional-dose strategies in patients with SpA.

Although the multivariable analysis accounted for previous SARS-CoV-2 infection, age, sex, vaccination regimen, disease subtype, and bDMARDs, residual confounding cannot be excluded. Therefore, the observed associations should be interpreted with caution, particularly given the observational nature of the study [31].

The ≥506 BAU/mL threshold was used solely as an exploratory reference. It was derived from a different study or for the vaccination regimens evaluated in the SAFER cohort. Therefore, the proportion of participants exceeding this threshold should not be interpreted as the proportion clinically protected against COVID-19.

Vaccination also demonstrated an excellent safety profile. Adverse events were predominantly mild, transient, and self-limited, and no vaccine-related SAEs or AESIs were identified. Disease activity remained stable throughout follow-up in both axSpA and PsA, with no clinically meaningful changes in ASDAS, BASDAI, PASI, or MDA. Although a small reduction in median ASDAS values was observed after additional dose vaccination, this finding should be interpreted cautiously because of the limited sample size and missing follow-up assessments.

The principal strengths of this study include its prospective multicenter design, real-world setting, standardized longitudinal assessment of disease activity, and direct comparison of different vaccine platforms and homologous versus heterologous vaccination strategies. The study also reflects the NIP, providing evidence that is directly applicable to routine clinical practice. Nevertheless, some limitations should be acknowledged. The relatively small sample size limited statistical power for subgroup analyses; no healthy control group was included, and neutralizing antibodies and cellular immune responses were also not evaluated.

Finally, the analysis was restricted to the first month after the additional dose; the durability of the post-booster humoral response and the need for additional doses could not be assessed.

In conclusion, SARS-CoV-2 vaccination was safe and highly immunogenic in patients with axSpA and PsA. Heterologous vaccination regimens incorporating an mRNA additional dose generated the strongest humoral responses without compromising disease control or safety. These findings reinforce current vaccination recommendations and provide additional real-world evidence supporting heterologous additional dose strategies for patients with SpA.

## 5. Conclusions

SARS-CoV-2 vaccination was safe and highly immunogenic in patients with axSpA and PsA, without clinically meaningful worsening of disease activity. Heterologous regimens incorporating a BNT162b2 additional dose elicited the strongest humoral immune responses. These findings provide prospective real-world evidence to inform future vaccination strategies and tailored immunization approaches for patients with SpA and other immune-mediated rheumatic diseases.

## Figures and Tables

**Table 1:** Immunosuppression level according to the Brazilian Society of Rheumatology (SBR).

Immunosuppression Level	Criteria According to the Brazilian Society of Rheumatology (SBR)
**no**	No use of immunosuppressive agents; exclusive use of antimalarial monotherapy (hydroxychloroquine or chloroquine); SSZ; topical, inhaled, or intra-articular corticosteroids
**low**	Prednisone ≤ 20 mg/day; MTX ≤ 0.4 mg/kg/week or ≤20 mg/week; AZA ≤ 3 mg/kg/day; leflunomide ≤ 20 mg/day
**high**	Prednisone ≥ 20 mg/day; glucocorticoid pulse therapy; cyclophosphamide; mycophenolate mofetil; cyclosporine; tacrolimus; bDMARDs (TNFi, IL-17i, IL-6i, IL-12/23i, IL-23i, abatacept, rituximab, among others); JAKi; or combination immunosuppressive therapy

AZA, azathioprine; bDMARDs, biologic disease-modifying antirheumatic drugs; IL, interleukin; IL-6i, interleukin-6 inhibitors; IL-17i, interleukin-17 inhibitors; IL-12/23i, interleukin-12/23 inhibitors; IL-23i, interleukin-23 inhibitors; JAKi, Janus kinase inhibitors; MTX, methotrexate; SSZ, sulfasalazine; TNFi, tumor necrosis factor inhibitors.

**Table 2:** Demographic, clinical, and treatment characteristics of patients with spondyloarthritis.

Characteristic	Total (N = 90)	Axial SpA (N = 69)	PsA (N = 21)	*p*
Age, years	44 [33–52]	41 [31–49]	52 [47–57]	<0.001
Sex				
Male	49 (54.0%)	42 (61.0%)	7 (33.0%)	0.027
Female	41 (46.0%)	27 (39.0%)	14 (67.0%)	
Skin color				
White	44 (49.0%)	34 (49.0%)	10 (48.0%)	>0.900
Non-White	46 (51.0%)	35 (51.0%)	11 (52.0%)	
Comorbidities				
Any comorbidity	40 (44.0%)	27 (39.0%)	13 (62.0%)	0.066
Cardiovascular disease	3 (3.3%)	3 (4.3%)	0 (0.0%)	
Hypertension	19 (21.0%)	10 (14.0%)	9 (43.0%)	
Diabetes mellitus	7 (7.8%)	4 (5.8%)	3 (14.0%)	
BMI category				
Underweight	1 (1.0%)	1 (1.0%)	0 (0.0%)	0.029
Normal weight	28 (31.0%)	25 (36.0%)	3 (14.0%)	
Overweight	35 (39.0%)	25 (36.0%)	10 (48.0%)	
Obesity	26 (29.0%)	18 (26.0%)	8 (38.0%)	
Lifestyle				
Current smoking	5 (5.6%)	3 (4.3%)	2 (9.5%)	0.300
Alcohol consumption	4 (4.4%)	4 (5.8%)	0 (0.0%)	0.600
Disease characteristics				
Disease duration, years	9.0 [4.0–14.0]	10.0 [4.0–14.0]	8.0 [5.0–11.0]	>0.900
HLA-B27 positive ^†^	34 (63.0%)	34 (76.0%)	0 (0.0%)	<0.001
Previous COVID-19	17 (19.0%)	12 (17.0%)	5 (24.0%)	0.500
Predominant musculoskeletal involvement ^§^				
Axial	73 (81.0%)	63 (91.0%)	5 (23.0%)	<0.001
Peripheral	27 (18.0%)	9 (9.0%)	16 (76.0%)	
Immunosuppression level				
Absent/Low	23 (26.0%)	20 (29.0%)	3 (14.0%)	0.200
Moderate/High	67 (74.0%)	49 (71.0%)	18 (86.0%)	
Current treatment exposure				
Daily NSAIDs	15 (17.0%)	15 (22%)	0 (0.0%)	0.018
Systemic glucocorticoids	2 (2.2%)	2 (2.9%)	0 (0.0%)	>0.900
Hydroxychloroquine	1 (1.1%)	0 (0.0%)	1 (4.8%)	0.233
csDMARDs				
Methotrexate	23 (26.0%)	13 (19.0%)	10 (48.0%)	0.020
Sulfasalazine	6 (6.7%)	5 (7.2%)	1 (4.8%)	>0.900
Leflunomide	4 (4.4%)	2 (2.9%)	2 (9.5%)	0.231
Cyclosporine	1 (1.1%)	0 (0%)	1 (4.8%)	0.233
bDMARDs				
TNFi	59 (66.0%)	46 (67%)	13 (62%)	0.794
IL-17i	4 (4.4%)	2 (2.9%)	2 (9.5%)	0.231
IL-23i	1 (1.1%)	0 (0.0%)	1 (4.8%)	0.233
tsDMARDs				
JAKi	1 (1.1%)	0 (0.0%)	1 (4.8%)	NE

^§^ patients could present both axial and peripheral manifestations; therefore, percentages do not sum to 100%. ^†^ percentage calculated among patients with available HLA-B27 results. Data are presented as *n* (%) or median [interquartile range]. *p*-values compare axSpA and PsA groups. axSpA: axial spondyloarthritis; PsA: psoriatic arthritis; BMI: body mass index; COVID-19: coronavirus disease 2019; NSAIDs: nonsteroidal anti-inflammatory drugs; csDMARDs: conventional synthetic disease-modifying antirheumatic drugs; bDMARDs: biologic disease-modifying antirheumatic drugs; tsDMARDs: targeted synthetic disease-modifying antirheumatic drugs; TNFi: tumor necrosis factor inhibitors; IL: interleukin; JAKi: Janus kinase inhibitors. NE: not estimable due to insufficient sample size.

**Table 3:** Comparison of anti-RBD IgG geometric mean titers between disease subtypes, vaccination regimens, previous SARS-CoV-2 infection status, and bDMARD therapy across study time points.

Comparison	T1 ^a^	T2 ^a^	T3 ^a^	T4 ^a^
PsA (n = 21) vs. axSpA (n = 69)	1.46 (0.49–4.35)	1.02 (0.32–3.26)	1.16 (0.56–2.39)	1.24 (0.65–2.37) *p* = 0.613
Previous SARS-CoV-2 infection (n = 31) vs. infection-naïve (n = 59)	NA	31.35 (15.18–64.74)	5.31 (3.09–9.11) *p* < 0.001	2.29 (1.33–3.97) *p* = 0.008
ChAdOx1-S (n = 58) vs. CoronaVac (n = 32) primary series	NA	9.98 (4.07–24.50) *p* < 0.001	3.93 (2.22–6.97) *p* < 0.001	NA
BNT162b2 (n = 55) vs. ChAdOx1-S (n = 35) additional-dose groups	NA	NA	0.42 (0.23–0.77) *p* = 0.015	3.62 (2.21–5.91) *p* < 0.001
Heterologous (n = 62) vs. homologous (n = 28) vaccination schedule	NA	NA	NA	3.92 (2.34–6.56) *p* < 0.001
bDMARD therapy (n = 66) vs. no bDMARD therapy (n = 24)	NA	NA	NA	1.44 (0.78–2.66) *p* = 0.226

^a^ values are GMT ratios (95% CI). *p*-values (Mann–Whitney U test). GMT, geometric mean titer; PsA, psoriatic arthritis; axSpA, axial spondyloarthritis; NA, not available; T1, before the first vaccine dose; T2, after the first dose; T3, after the second dose and before the additional dose; T4, after the additional dose.

**Table 4:** Longitudinal humoral response to SARS-CoV-2 vaccination according to disease subtype and primary vaccination regimen.

Time	Total	Axial SpA	PsA	*p*	CoronaVac	ChAdOx1-S	*p*
**T1**	6.70 [4.24–10.60] ^†^	6.13 [3.70–10.16] ^†^	8.98 [2.89–27.85] ^†^	0.500	6.17 [2.79–13.64] ^†^	7.01 [3.93–12.52] ^†^	>0.900
**T2**	87.58 [53.87–142.41] ^†^	87.09 [50.72–149.55] ^†^	89.23 [27.35–291.10] ^†^	0.900	19.88 [9.83–40.23] ^†^	198.48 [114.22–344.90] ^†^	<0.001
**T3**	207.27 [152.77–281.20] ^†^	200.28 [143.96–278.63] ^†^	232.00 [105.46–510.35] ^†^	0.800	85.75 [54.74–134.30] ^†^	337.30 [237.24–479.57] ^†^	<0.001
**T4**	907.72 [691.41–1191.69] ^†^	863.24 [627.05–1188.38] ^†^	1070.65 [617.89–1855.17] ^†^	0.600	—	—	—
**Fold Increase ^‡^**							
**T1–T2**	—	—	—	—	3.22 [1.18–8.81] ^‡^	28.30 [13.42–59.68] ^‡^	<0.001
**T2–T3**	—	—	—	—	4.30 [1.95–9.55] ^‡^	1.70 [1.13–2.57] ^‡^	0.002

^†^ data are presented as binding antibody units per milliliter (BAU/mL), geometric mean titers [GMTs] with 95% confidence intervals [95% CI]; ^‡^ fold increase (FI) was calculated as the ratio of antibody titers between consecutive time points (T2/T1 and T3/T2) and is presented as median [interquartile range]. T1: pre-vaccination; T2: before the second vaccine dose; T3: before the additional dose; T4: 4 weeks after the additional dose. axSpA: axial spondyloarthritis; PsA: psoriatic arthritis.

**Table 5:** Seroresponse rates following SARS-CoV-2 vaccination in patients with axial spondyloarthritis and psoriatic arthritis, overall and by clinical and vaccination characteristics.

Group	T1	T2	T3	T4
**Overall**	31/90 (34.4%; 25.4–44.7)	72/90 (80.0%; 70.6–87.0)	90/90 (100.0%; 95.9–100.0)	90/90 (100.0%; 95.9–100.0)
**No previous SARS-CoV-2 infection**	0/59 (0.0%; 0.0–6.1)	41/59 (69.5%; 56.9–79.7)	59/59 (100.0%; 93.9–100.0)	59/59 (100.0%; 93.9–100.0)
**axSpA**	23/69 (33.3%; 23.4–45.1)	56/69 (81.2%; 70.4–88.6)	69/69 (100.0%; 94.7–100.0)	69/69 (100.0%; 94.7–100.0)
**PsA**	8/21 (38.1%; 20.8–59.1)	16/21 (76.2%; 54.9–89.4)	21/21 (100.0%; 84.5–100.0)	21/21 (100.0%; 84.5–100.0)
**CoronaVac** **primary series**	10/32 (31.2%; 18.0–48.6)	17/32 (53.1%; 36.4–69.1)	32/32 (100.0%; 89.3–100.0)	32/32 (100.0%; 89.3–100.0)
**ChAdOx1-S** **primary series**	21/58 (36.2%; 25.1–49.1)	55/58 (94.8%; 85.9–98.2)	58/58 (100.0%; 93.8–100.0)	58/58 (100.0%; 93.8–100.0)
**ChAdOx1-S** **additional dose**	16/35 (45.7%; 30.5–61.8)	33/35 (94.3%; 81.4–98.4)	35/35 (100.0%; 90.1–100.0)	35/35 (100.0%; 90.1–100.0)
**BNT162b2** **additional dose**	15/55 (27.3%; 17.3–40.2)	39/55 (70.9%; 57.9–81.2)	55/55 (100.0%; 93.5–100.0)	55/55 (100.0%; 93.5–100.0)

Data are presented as the number of participants with a serological response divided by the number evaluated (n/N), followed by the percentage and 95% confidence interval (CI), calculated using the Wilson method. Seroresponse was defined as an anti–SARS-CoV-2 receptor-binding domain (anti-RBD) IgG concentration ≥7.1 BAU/mL. T1, before the first vaccine dose; T2, before the second dose; T3, before the additional dose; T4, 28 days after the additional dose. Previous infection refers to SARS-CoV-2 infection before vaccination. Primary vaccination regimen refers to the vaccine received for the first two doses; additional-dose vaccine refers to the vaccine received as the third dose. axSpA, axial spondyloarthritis; PsA, psoriatic arthritis; BAU/mL, binding antibody units per milliliter; CI, confidence interval.

**Table 6:** Longitudinal comparison of geometric mean titers according to each additional dose.

Time Point	ChAdOx1-S (n = 35)	BNT162b2 (n = 55)	*p* ^2^
T3 ^†^	352.62 [217.00–572.98]	147.80 [101.43–215.37]	0.015
T4 ^†^	413.68 [257.12–665.56]	1496.70 [1154.50–1940.31]	<0.001
Fold Increase (T3–T4)	1.17 [0.80–1.71]	10.13 [7.28–14.09]	<0.001

^†^ data are presented as geometric mean titers (GMTs) [95% confidence interval]. ^2^ comparisons between groups were performed using the Mann–Whitney U test. Fold increase was calculated as the ratio between antibody titers at T4 and T3.

**Table 7:** Disease activity during follow-up according to PGA, ASDAS, BASDAI, PASI, and MDA.

Variable	Category	T1	T2	T3	T4	*p* *
PGA-M (N = 90)	No/low disease activity	49 (54.4%)	48 (53.3%)	64 (71.1%)	53 (58.8%)	0.030
	Moderate/high disease activity	41 (45.6%)	29 (32.2%)	25 (27.8%)	24 (26.7%)	
	Missing data	0 (0.0%)	13 (14.4%)	1 (1.1%)	13 (14.4%)	
	Median [IQR]	4.00 [1.00–6.00]	3.00 [2.00–5.00]	2.00 [1.00–5.00]	2.00 [1.00–5.00]	
ASDAS (N = 23)	Inactive/low disease activity	13 (56.5%)	7 (30.4%)	10 (43.5%)	13 (56.5%)	0.180
	High/very high disease activity	10 (43.5%)	5 (21.7%)	6 (26.1%)	2 (8.7%)	
	Missing data	0 (0.0%)	12 (%)	7 (7%)	8 (34.7%)	
	Median [IQR]	1.77 [0.97–3.51]	2.07 [1.74–2.66]	1.34 [0.78–2.96]	1.00 [0.93–1.40]	
BASDAI (N = 69)	<4	37 (53.6%)	29 (42.0%)	41 (59.4%)	35 (50.7%)	0.210
	≥4	25 (36.2%)	25 (36.2%)	24 (34.8%)	18 (26.1%)	
	Missing data	7 (10.1%)	15 (21.7%)	4 (5.8%)	16 (23.2%)	
	Median [IQR]	3.35 [1.13–4.96]	3.38 [1.20–5.15]	3.00 [1.20–5.10]	2.60 [1.00–4.15]	
PASI (N = 21)	<10	8 (38.1%)	6 (28.6%)	11 (52.4%)	6 (28.6%)	0.740
	≥10	13 (61.9%)	15 (71.4%)	10 (47.6%)	15 (71.4%)	
MDA (N = 21)	Achieved MDA	14 (66.7%)	10 (47.6%)	8 (38.1%)	12 (57.1%)	0.720
	Not achieved MDA	7 (33.3%)	9 (42.9%)	12 (57.1%)	6 (28.6%)	
	Missing data	0 (0.0%)	2 (9.5%)	1 (4.8%)	3 (14.3%)	

* *p*-values compare disease activity distributions across study time points (T1–T4). Abbreviations: PGA-M: Physician Global Assessment; ASDAS: Ankylosing Spondylitis Disease Activity Score; BASDAI: Bath Ankylosing Spondylitis Disease Activity Index; PASI: Psoriasis Area and Severity Index; MDA: Minimal Disease Activity.

**Table 8:** Factors associated with Anti-RBD IgG concentrations at T4: univariable and multivariable linear regression analyses.

Variable	Univariable exp(β) (95% CI)	*p*-Value	Multivariable exp(β) (95% CI)	*p*-Value
BNT162b2 additional dose(vs. ChAdOx1-S)	3.62 (2.21–5.91)	<0.001	4.34 (2.71–6.95)	<0.001
ChAdOx1-S primary series (vs. CoronaVac)	0.60 (0.34–1.06)	0.076	0.84 (0.52–1.35)	0.466
Previous SARS-CoV-2 infection	2.29 (1.33–3.97)	0.003	3.23 (2.01–5.19)	<0.001
Age (per 10-year increase)	1.04 (0.82–1.31)	0.773	0.96 (0.79–1.18)	0.708
Female sex	1.18 (0.68–2.03)	0.560	1.62 (1.00–2.63)	0.050
PsA (vs. axSpA)	1.24 (0.65–2.37)	0.509	0.93 (0.53–1.64)	0.799
**bDMARD** therapy	1.44 (0.78–2.66)	0.242	1.29 (0.75–2.22)	0.360

Values are presented as exponentiated regression coefficients [exp(β)] with 95% CIs, obtained from linear regression models of log-transformed anti-RBD IgG at T4. The multivariable model included all variables listed in the table (n = 90; adjusted R^2^ = 0.38). Age was analyzed per 10-year increase. All *p*-values are two-sided. CI, confidence interval; IgG, immunoglobulin G; RBD, receptor-binding domain; PsA, psoriatic arthritis; axSpA, axial spondyloarthritis; T4, post-additional-dose study time point.

**Table 9:** Local and systemic adverse events following the primary SARS-CoV-2 vaccination schedule and additional dose in patients with spondyloarthritis.

Adverse Event	Primary Vaccination				Additional Dose			
	CoronaVac (n = 32)	ChAdOx1-S (n = 58)	*p*	Risk Difference ^b^ (IC)	ChAdOx1-S (n = 35)	BNT162b2 (n = 55)	*p*	Risk Difference ^b^ (IC)
**Local Reactions**								
**Erythema**	4 (13.0%)	10 (17.0%)	0.800		0/31 (0.0%)	7/47 (15.0%)	0.038	+14.9 (+1.6 ^a^ +27.7)
**Ecchymosis**	3 (9.4%)	11 (19.0%)	0.400		0/31 (0.0%)	3/47 (6.4%)	0.300	
**Local skin lesion**	6 (19.0%)	5 (8.6%)	0.200		2/31 (6.5%)	1/47 (2.1%)	0.600	
Pruritus *	4/6 (67.0%)	3/5 (60.0%)	>0.990		1/2 (50.0%)	0/1 (0.0%)	>0.900	
**Swelling**	4 (13.0%)	17 (29.0%)	0.070		1/31 (3.2%)	10/47 (21.0%)	0.040	+18.1 (+ 2.1 ^a^ +31.9)
**Local induration**	5 (16.0%)	19 (33.0%)	0.078		3/31 (9.7%)	9/47 (19.0%)	0.300	
**Injection-site pain**	12 (38.0%)	46 (79.0%)	<0.001	+41.8 (+20.8 ^a^ +58.6)	15/31 (48.0%)	31/47 (66.0%)	0.120	
**Systemic Reactions**								
**Nausea/Vomiting**	8 (25.0%)	15 (26.0%)	>0.990		3/31 (9.7%)	6/47 (13.0%)	>0.900	
**Fatigue**	8 (25.0%)	31 (53.0%)	0.009	+28.4 (+7.2 ^a^ +45.4)	8/31 (26.0%)	9/47 (19.0%)	0.500	
**Headache**	11 (34.0%)	34 (59.0%)	0.028		7/31 (23.0%)	7/47 (15.0%)	0.400	
**Myalgia**	11 (34.0%)	29 (50.0%)	0.200		8/31 (26.0%)	11/47 (23.0%)	0.800	
**Arthralgia**	12 (38.0%)	34 (59.0%)	0.055		12/31 (39.0%)	14/47 (30.0%)	0.400	
**Fever**	6 (19.0%)	20 (34.0%)	0.110		4/31 (13.0%)	2/47 (4.3%)	0.200	
**Dizziness/Vertigo**	8 (25.0%)	13 (22.0%)	0.800		4/31 (13.0%)	2/47 (4.3%)	0.200	
**Other systemic** **symptoms**	7 (22.0%)	20 (34.0%)	0.200		2/31 (6.5%)	5/47 (11.0%)	0.700	
**Overall Adverse Events**								
**≥1 local adverse event**	14 (44.0%)	48 (83.0%)	<0.001	+39.0 (+18.5 ^a^ +56.3)	17/31 (55.0%)	34/47 (72.0%)	0.110	
**≥1 systemic adverse event**	18 (56.0%)	44 (76.0%)	0.054		17/31 (55.0%)	21/47 (45.0%)	0.400	

Data are presented as n (%). ^b^ Risk difference was calculated as the difference in the proportion of participants with the event between the two groups, ^a^ risk differences for adverse events (Newcombe method) (IC 95%). * pruritus was assessed only among participants reporting local skin lesions. Values are presented as n/N (%) unless otherwise indicated. Risk differences are expressed in percentage points with 95% confidence intervals calculated using the Newcombe method. For primary vaccination, risk differences were calculated as ChAdOx1-S minus CoronaVac; for the additional dose, as BNT162b2 minus ChAdOx1-S. *p*-values were calculated using Fisher’s exact test. Denominators reflect the number of participants with available safety data for each vaccination period.

## Data Availability

The data presented in this study are not publicly available due to ethical and privacy restrictions related to the protection of participant confidentiality. De-identified data may be made available by the corresponding author upon reasonable request and subject to approval by the coordinating investigators and the relevant Research Ethics Committees, in accordance with institutional and national regulations.

## Abbreviations

The following abbreviations are used in this manuscript:

Abbreviation	Definition
ACR	American College of Rheumatology
ANVISA	Brazilian Health Regulatory Agency
ASAS	Assessment of SpondyloArthritis International Society
axSpA	Axial Spondyloarthritis
BASDAI	Bath Ankylosing Spondylitis Disease Activity Index
bDMARDs	Biologic Disease-Modifying Antirheumatic Drugs
CASPAR	Classification Criteria for Psoriatic Arthritis
csDMARDs	Conventional Synthetic Disease-Modifying Antirheumatic Drugs
DECIT	Department of Science and Technology, Brazilian Ministry of Health
DMARDs	Disease-Modifying Antirheumatic Drugs
EULAR	European Alliance of Associations for Rheumatology
IMRDs	Immune-Mediated Rheumatic Diseases
JAKi	Janus Kinase Inhibitor(s)
MDA	Minimal Disease Activity
NSAID	Nonsteroidal Anti-Inflammatory Drug
PASI	Psoriasis Area and Severity Index
PGA	Physician Global Assessment
PNI	Brazilian National Immunization Program
PsA	Psoriatic Arthritis
RT-PCR	Reverse Transcription Polymerase Chain Reaction
SBR	Brazilian Society of Rheumatology (Sociedade Brasileira de Reumatologia)
SpA	Spondyloarthritis
TNFi	Tumor Necrosis Factor Inhibitor(s)
tsDMARDs	Targeted Synthetic Disease-Modifying Antirheumatic Drugs
